# Percutaneous Thrombin Injection with Balloon Protection for a Large Iatrogenic Femoral Artery Pseudoaneurysm: A Case Report with Review of Literature

**DOI:** 10.15388/Amed.2023.30.1.9

**Published:** 2023-05-16

**Authors:** Ranjan Patel, Tara Prasad Tripathy, Ripan Debbarma, Sudipta Mohakud, Satyapriya Mohanty, Nerbadyswari Deep Bag

**Affiliations:** Department of Radiodiagnosis, AIIMS, Bhubaneswar, India; Department of Radiodiagnosis, AIIMS, Bhubaneswar, India; Department of Radiodiagnosis, AIIMS, Bhubaneswar, India; Department of Radiodiagnosis, AIIMS, Bhubaneswar, India; Department of CTVS, AIIMS, Bhubaneswar, India; Department of Radiodiagnosis, AIIMS, Bhubaneswar, India

**Keywords:** Percutaneous thrombin injection, femoral artery pseudoaneurysm, ballon assisted

## Abstract

Iatrogenic femoral artery pseudoaneurysms (IFAPs) are not uncommon due to the increase in various minimally-invasive endovascular procedures. Percutaneous thrombin injection is an established technique for large pseudoaneurysms. When ultrasound-guided compression of an aneurysmal neck is not feasible, percutaneous thrombin injection can be combined with endovascular balloon occlusion to prevent leakage of thrombin into the parent artery. We describe a large IFAP following the removal of the femoral dialysis catheter after an inadvertent arterial puncture, which was managed with percutaneous ultrasound-guided (USG) thrombin injection with simultaneous balloon occlusion at the level of the aneurysmal neck without any complications. Follow-up imaging showed thrombosed IFAP without any recurrence.

## Santrauka.

Jatrogeninės femoralinės arterijos pseudoaneurizmos (JFAP) nėra retos, nes gausėja įvairių minimaliai invazyvių endovaskulinių procedūrų. Perkutaninė trombino injekcija yra paplitusi didelių pseudoaneurizmų atveju taikoma gydymo technika. Kai neįmanoma pritaikyti ultragarsu nukreipiamo aneurizminio kaklelio užspaudimo, galima derinti perkutaninę trombino injekciją ir endovaskulinio baliono okliuziją, kad būtų išvengta trombino nuotėkio į motininę arteriją. Aprašome didelę JFAP, atsiradusią išimant femoralinės dializės kateterį ir netyčia persmeigus arteriją. Problema buvo išspręsta atliekant perkutaninę ultragarsu nukreipiamą trombino injekciją ir tuo pačiu metu ties aneurizmos kakleliu atliekant baliono okliuziją. Jokių komplikacijų neįvyko. Stebėjimo laikotarpiu užfiksuota, kad trombinė JFAP nebepasikartojo.

## Introduction

Iatrogenic femoral artery pseudoaneurysms (IFAPs) are mostly encountered following femoral artery catheterization, required for various therapeutic and diagnostic endovascular procedures. Although the incidence of IFAP ranges from 1.1 to 7.7%, the proportion of symptomatic IFAP is relatively low.^1, 2^ Common symptoms of femoral artery pseudoaneurysm (PSA) include pain, pulsatile swelling, and local bruising. The most devastating complication is rupture which mainly depends on the size of the PSA. Other complications include persistent pain and swelling, distal embolization, local site skin ischemia and necrosis, infection, compressive vasculopathy, and neuropathy.^3^

Depending on the symptoms, location, and morphology of the PSA, treatment options vary, ranging from ultrasound-guided compression therapy (UGCT), simple percutaneous injection of tissue adhesives to most invasive approaches such as coil embolization, stent-graft placement, or open surgical repair.^3, 4^ Here we describe a case of large femoral artery PSA following the removal of the right femoral dialysis catheter after an inadvertent arterial puncture, which was successfully managed by percutaneous thrombin injection with simultaneous endovascular balloon occlusion to avoid spillage of thrombin into the parent artery.

## Case report

**Figure 1:**
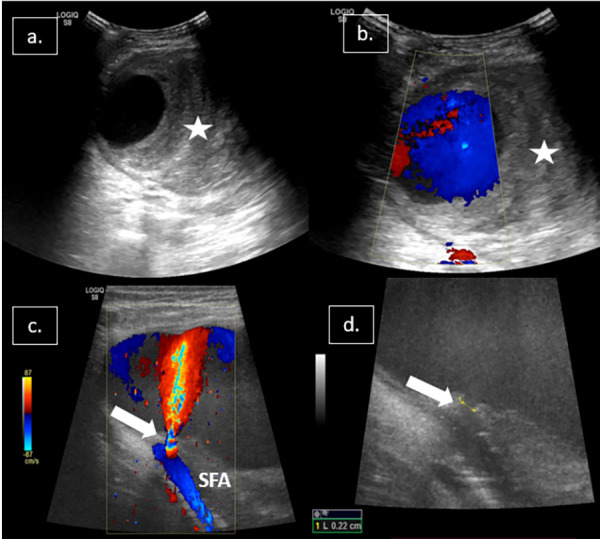
(a, b) USG shows a large pseudoaneurysm (PSA) with to and fro flow on color Doppler within the aneurysmal sac. Eccentric echogenic thrombus (white star) is noted inside the PSA; (c) Doppler showing jet-like flow from SFA into the PSA sac; (d) PSA neck measures 2.2 mm.

A 42-year-old man with end-stage renal disease (ESRD) on maintenance hemodialysis presented to the outpatient department with complaints of a gradually increasing pulsatile and painful swelling at the right groin for 15 days. On examination, a soft and pulsatile swelling of 5.0 × 4.0 × 3.0 cm size was palpated, which was compressible and tender. Local ecchymosis was also noted over the swelling. Otherwise, the patient was hemodynamically stable with a pulse rate of 88 beats/min and blood pressure of 148/82 mm Hg. Hemoglobin was 8.2 gm/dl. INR and platelet counts were 1.3 and 2.1 lakhs/mm^3^, respectively. Remaining of the lab parameters were within the normal ranges. On detailed elicitation of history, the patient noticed the swelling after removing the right femoral dialysis catheter, indicating inadvertent arterial puncture. Detailed ultrasonography (US) revealed an oval-shaped anechoic cystic lesion (5.4 × 3.2 × 2.5 cm) in the right groin with peripheral eccentric hematoma (Fig. 1a, 1b). Color Doppler showed a jet-like flow from the superficial femoral artery into the anechoic sac with the turbulent flow within the anechoic sac, suggesting a pseudoaneurysm. The neck of the PSA measured 2.2 mm in diameter (Fig. 1b-1d).

To treat the PSA, USG-guided percutaneous thrombin injection combined with simultaneous endovascular balloon protection to prevent thrombin leakage was planned since the large size of the PSA and tenderness prevented effective compression of the PSA neck. The written informed consent was taken from the patient and this case report was approved by the our multidisciplinary committee.

## Technique

**Figure 2:**
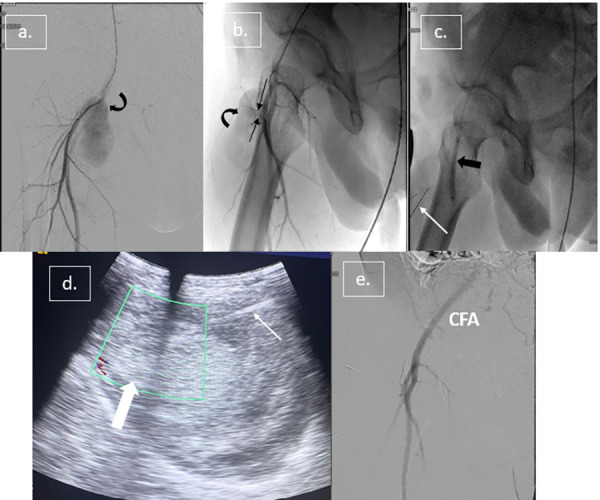
(a, b) Angiographic run shows a contrast-filled oval-shaped pseudoaneurysm (curved black arrow) arising from SFA as indicated by jet-like neck (thin black arrows); (c) 2 LP needles (21G) were placed within PSA (white arrow c, d), and then 8 mm balloon (thick black arrow) was inflated across the neck of PSA; (d) USG-guided thrombin injection showing thrombosis of PSA sac (thick white arrow); (e) Post-embolization DSA shows nonopacification of PSA with normal opacification of SFA

Under local anesthesia, left femoral access was obtained under USG guidance, and a 6F cross-over vascular sheath (Balkin sheath, Cook Medical) was placed. Digital subtraction angiography (DSA) through a 5F multipurpose (MPA) catheter demonstrated a large oval-shaped contrast-filled pseudoaneurysm arising from the superficial femoral artery (SFA) just distal to the bifurcation of the common femoral artery (Fig. 2a, 2b). Then, an 8 x 60 mm balloon (Mustang balloon catheter, Boston Scientific) was inflated temporarily in the SFA across the PSA neck. Temporary balloon inflation for 30 seconds showed remarkable reduction in intra-aneurysmal flow on Doppler imaging (Fig. 2c).

Two 21G lumbar puncture (LP) needles were inserted into the PSA near the dome under the USG guidance, with the tip of one needle towards the right and the other towards the left side of the PSA (Fig. 2d). The free flow of blood through the needle hub confirmed the correct positioning. The balloon was inflated under the fluoroscopy and 2500 IU of heparin was administered into the distal arteries through the balloon catheter. After that under the real-time USG guidance, 1.5 ml of reconstituted thrombin solution was administered slowly through each LP needle simultaneously using 2 ml syringes (Fig. 2e). USG following 8 minutes of thrombin injection showed thrombosis of most of the PSA except for a small area near the PSA neck showing persistent flow. The balloon was deflated temporarily for 2 min to resume distal blood flow. Again balloon was inflated, one of the needles was repositioned near the PSA neck, and 1 ml of thrombin solution was administered further under balloon occlusion. USG, after 5 mins, showed complete thrombosis of PSA without any residual flow. The angiographic run also showed nonopacification of the PSA. No embolism was observed in the SFA or distal arteries of the right lower extremity (Fig. 2f). LP needles were removed, and left-sided femoral access site hemostasis was achieved with local compression after the sheath removal. A total of 5000 IU of IV heparin was administered during the procedure to prevent thromboembolic complications. The patient was kept on bed rest for 6 hours after the procedure.

**Figure 3:**
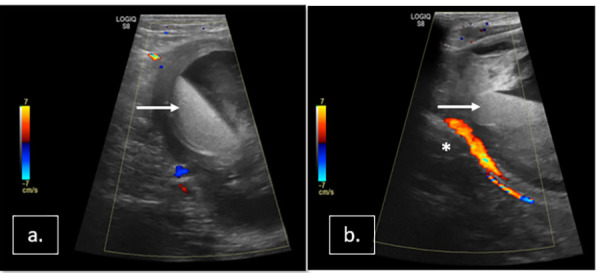
(a, b) Follow-up USG showing thrombosed PSA with no flow on Doppler imaging. Thrombosed PSA sac showing dependent echogenic retracted clot (white arrow) and nondependent anechoic serum.

Due to nonavailability of recombinant human thrombin recommended for endovascular use, FloSeal kit (Baxter) was used in our case. Of note, only recombinant human thrombin component with CaCl_2_ was used in our case excluding the gelatin matrix component of the FloSeal kit. Lyophilized human thrombin powder (2500 IU) was mixed with 5 ml of Calcium Chloride solution and the reconstituted solution was drawn into a 5 ml syringe.

Follow up:

Follow-up USG with color Doppler on day 1 and 25 demonstrated thrombosed PSA (Fig. 3) and patent SFA, anterior and posterior tibial arteries, confirming no obvious distal embolization.

## Discussion

Various risk factors for post-catheterization pseudoaneurysm (PCPA) include obesity, anticoagulation, hypertension, heavily calcified arteries, hemodialysis, large sheath size (>7F), faulty puncture, simultaneous catheterization of artery and vein, brief manual compression, and difficult to compression sites.^3^ Our patient had chronic kidney disease (CKD) and was on maintenance hemodialysis. Additionally, there was an inadvertent arterial puncture during dialysis catheter insertion and that of the superficial femoral artery, which poses a higher risk of pseudoaneurysm.^3, 4^

USG with Doppler is the imaging of choice in the case of PCPA. Doppler shows turbulent flow within the sac, also called the “yin-yang sign”, while spectral Doppler shows the arterial waveform^1-3^. However, CT or MR angiography may be required for detailed vascular mapping before planning a therapeutic procedure.^1, 3^

Most femoral PSAs are managed with minimally invasive approaches except for a few indications that require surgery. Ultrasound-guided thrombin injection is a simple and safe technique to treat large IFAP.^4^ Theoretically, a long and narrow neck is associated with a decreased risk, while a short and wide neck poses an increased risk of thrombin leakage into the parent artery.^3-5^ In our case, large aneurysmal size and tenderness precluded effective neck compression; hence, thrombin injection under balloon protection was planned to prevent thromboembolism. Stent-graft placement could be an option; however, the location of arterial lesion and high cost rendered its use unreasonable.

Combined use of parent artery balloon occlusion and embolic agent administration of PSAs has been reported previously.^5-13^ Samal et al. successfully treated 4 cases of IFAP with percutaneous thrombin injection with balloon protection without any complications or recurrence. Reasons not suitable for USG-guided compression therapy (UGCT) included severe vascular disease, ongoing anticoagulation, and large size and tenderness of IFAP.^6^ Nakai et al. used glue (N-butyl cyanoacrylate) and lipiodol mixture in place of thrombin to embolize 3 cases of IFAP under balloon protection.^7^

In a published case series of 25 PSAs by Owen et al., 19 were successfully embolized with tissue adhesive (fibrin glue/thrombin) combined with protective balloon occlusion, although one patient developed access site IFAP.^8^

In a recent study, Hayakawa et al. demonstrated the efficacy and safety of the percutaneous thrombin injection combined with endovascular balloon occlusion for IFAP. Their study included 11 cases of IFAP. None of the patients developed any complications.^9^

Embolization of upper extremity PSA using this technique has also been reported.^10,11^ In addition, Holder et al. reported a case of carotid artery PSA treated successfully with percutaneous thrombin injection combined with transient balloon occlusion of the common carotid artery for 10 sec. No neurological complication was observed.^12^

Of note, this technique may be complicated by contralateral puncture site problems, parent artery rupture or dissection secondary to balloon inflation, distal embolism, and PSA rupture ^5-13^. Bhat et al. reported a case of IFAP that was complicated by SFA thrombosis despite using balloon protection while embolizing with thrombin.^13^

Although our case was effectively managed by percutaneous thrombin injection under balloon occlusion. However prospective studies involving a large sample size are needed to establish the safety and efficacy of this technique.

## Conclusions

When UGCT fails or is not feasible, USG-guided thrombin injection combined with simultaneous balloon inflation across the neck provides a safe and effective option to tackle large IFAPs. However, the potential risks should be considered while using this technique.
